# Ultrasound settings significantly alter arterial lumen and wall thickness measurements

**DOI:** 10.1186/1476-7120-6-6

**Published:** 2008-01-22

**Authors:** Kathleen Potter, Christopher J Reed, Daniel J Green, Graeme J Hankey, Leonard F Arnolda

**Affiliations:** 1 School of Medicine and Pharmacology, University of Western Australia, Perth, Australia; 2 Department of Medical Engineering and Physics, Royal Perth Hospital, Perth, Australia; 3 Research Institute for Sport and Exercise Science, Liverpool John Moores, University, Liverpool, UK; 4 School of Sport Science, Exercise and Health, The University of Western, Australia, Perth, Australia; 5 Department of Neurology, Royal Perth Hospital, Perth, Australia; 6 Department of Cardiology, Royal Perth Hospital, Perth, Australia

## Abstract

**Background:**

Flow-mediated dilation (FMD) and carotid intima-medial thickness (CIMT), measured by ultrasound, are widely used to test the efficacy of cardioprotective interventions. Although assessment methods vary, automated edge-detecting image analysis software is routinely used to measure changes in FMD and CIMT. We aimed to quantify the effect that commonly adjusted ultrasound settings have on arterial lumen and wall thickness measurements made with CIMT measurement software.

**Methods:**

We constructed phantom arteries from a tissue-mimicking agar compound and scanned them in a water bath with a 10 MHz multi-frequency linear-array probe attached to a high-resolution ultrasound machine. B-mode images of the phantoms were recorded with dynamic range (DR) and gain set at five decibel (dB) increments from 40 dB to 60 dB and -10 dB to +10 dB respectively. Lumen diameter and wall-thickness were measured off-line using CIMT measurement software.

**Results:**

Lumen measurements: there was a strong linear relationship between DR and gain and measured lumen diameter. For a given gain level, a 5 dB increase in DR reduced the measured lumen diameter by 0.02 ± 0.004 mm (p < 0.001). For a given DR level, a 5 dB increase in gain reduced measured lumen diameter by 0.04 ± 0.004 mm (p < 0.001). A 5 mm increase in distance between the ultrasound probe and the artery reduced measured lumen diameter by 0.04 ± 0.03 mm (p < 0.001)

CIMT measurements: For a fixed gain level, a 5 dB increase in DR increased measured wall thickness by 0.003 ± 0.002 mm (p < 0.001). The effects of increasing gain were not consistent and appeared to vary depending on the distance between the artery and the ultrasound probe and the thickness of the artery wall.

**Conclusion:**

DR, gain and probe distance significantly alter lumen diameter and CIMT measurements made using image analysis software. When CIMT and FMD are used to test the efficacy of cardioprotective interventions, the DR, gain and probe position used to record baseline scans should be documented and replicated in post-treatment scans in individual trial subjects. If more than one sonographer or imaging centre is used to collect data, the study protocol should document specific DR and gain settings to be used in all subjects.

## Background

Carotid intima-medial thickness (CIMT) and flow-mediated dilation (FMD) are widely accepted as indicators of early atherosclerotic change [1,2]. CIMT and FMD are both measured using transcutaneous ultrasound: CIMT is the distance between the lumen-intima and media-adventitia interfaces on a B-mode image of the carotid artery and FMD is the increase in brachial artery diameter in response to an ischemic stimulus [3,4]. Both measurements correlate well with clinical endpoints [5-7] and are assessed using a safe and non-invasive imaging modality. Consequently CIMT and FMD are frequently used as surrogates for vascular events in intervention studies, and investigators often report small but statistically significant changes in FMD or CIMT as evidence that an intervention alters cardiovascular risk [8-12] However FMD and CIMT are subject to multiple sources of measurement error that, unless controlled or accounted for, may make such results unreliable [13].

Arterial wall thickness and lumen diameter are commonly measured with edge-detecting image analysis software. Image analysis software typically requires the arterial wall echoes to be bright and the lumen to be dark and free of noise in order for the edge-detection algorithm to identify the echo lines correctly. When the ultrasound scans are recorded, sonographers adjust the ultrasound settings of gain (regulates the brightness of the image by amplifying echoes) and/or dynamic range (DR, controls the contrast of the image and also known as log-compression) to optimise the images for later off-line analysis [14]. Lumen diameter is known to be underestimated when measured with intravascular ultrasound, and increasing gain and DR magnifies the error[15] Transcutanous ultrasound is used to assess FMD and CIMT and the effect of adjusting ultrasound parameters on these measurements has not been quantified. We aim to determine whether adjusting DR and gain alters the calculation of arterial lumen diameter and wall thickness, assessed with CIMT measurement software on B-mode ultrasound images.

## Methods

We tested the effect of altering DR and gain with artificial tissue-mimicking "phantom" arteries rather than human subjects. Using phantoms allowed us to construct vessels with known dimensions, eliminate movement, remove biological sources of variation and control several other variables such as probe position, temperature and region of interest selection.

### Phantom artery construction

**Figure 1:**
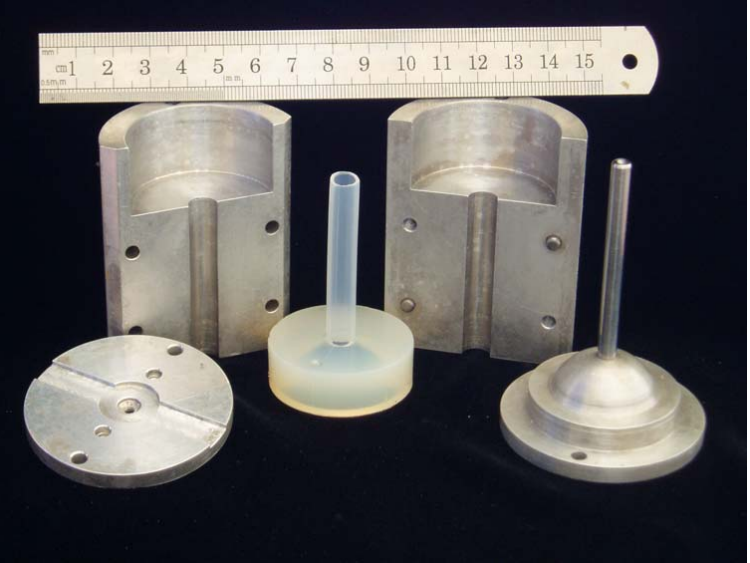
**Mold used to construct phantom arteries**. Aluminum mold used to construct phantom arteries showing the male and female parts and an agar phantom artery constructed using the mold.

We constructed phantom arteries using materials and methods similar to those described by Brunette [16]. We machined two aluminium molds to a precision of ± 0.1 mm. Each mold had a male part, two female parts and a base designed to centre the male part in the female (Figure 1). The male parts were designed to have external diameters of 5 mm and 6 mm and the female parts to have internal diameters of 6 mm and 8 mm. We used different combinations of male and female parts to make arteries with wall-thicknesses of approximately 0.5 mm, 1 mm or 1.5 mm and lumen diameters of 5 mm and 6 mm.

The phantom arteries were constructed from 5 ml of glycerol (Sigma-Aldrich, St Louis, MO, USA, C789-3), 8 g of high strength agar gel (Sigma Chemical, St Louis, MO, A-6924) and 150 ml of water. These constituents were mixed well, heated in a water bath at 100°C for 60 minutes and then poured into a pre-heated female mold. The male part was forced into the female and held position with screws. The mold was allowed to cool and we removed the base and separated the male and female parts to extract the phantom. Phantom arteries made from this compound have acoustic properties that are virtually identical to tissue [16-18].

### Recording the B-mode ultrasound images

**Figure 2:**
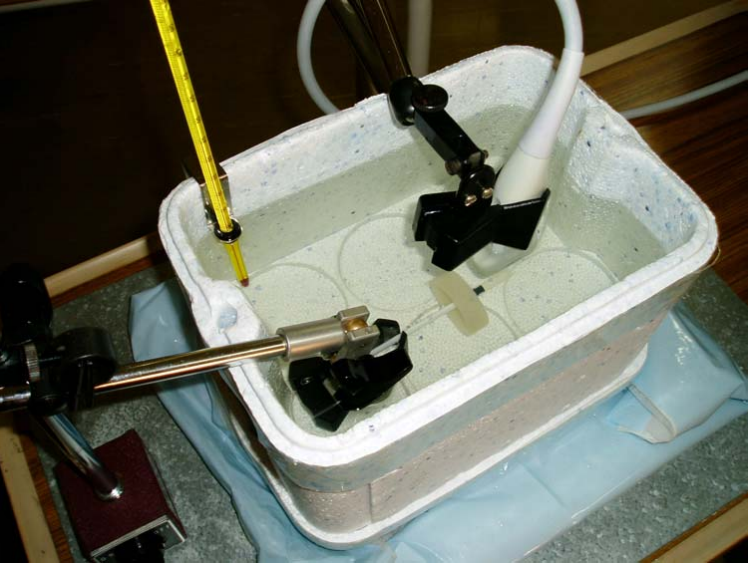
**Scanning set-up for recording phantom images**. Phantom and probe held in fixed position with stereotactic clamps in a water bath at approximately 37°C.

The phantom arteries were scanned in an insulated container of water at approximately 37°C. The phantom and the ultrasound probe were held in place with stereotactic clamps (Figure 2). The exam and image presets (persistence, edge-tracking and pre- and post-processing) used for human vascular imaging were used for the phantom scans. Early results showed that the distance between the probe and the phantom influenced lumen measurements, so we recorded scans with the probe set at 10 mm, 15 mm, 20 mm and 25 mm from the phantom. At each distance setting, the transmit zone (focal zone) was set as close as possible to the far wall of the artery. Depth gain compensation (DGC, also known as time gain compensation) was adjusted with the image at DR 50 dB and gain 0 dB to ensure that the near and far walls of the artery were of similar brightness and that the lumen was dark. The DGC was not altered again during the scan. For each distance setting, we adjusted the DR in 5 dB increments from 40 to 60 dB. For each DR setting, we adjusted the overall gain in 5 dB increments from -10 to +10 dB and recorded each image for approximately 2 seconds. Early results suggested that the phantoms were not completely symmetrical, so we repeated these sets of scans four times for each phantom, rotating the phantom a quarter-turn for each set of recordings. Final values were averaged from the results from each of these four positions.

**Figure 3:**
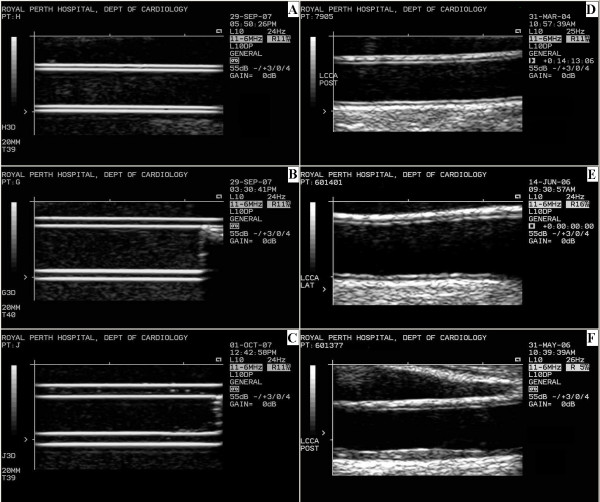
**B-mode ultrasound images of phantom arteries**. A. Phantom A with a wall thickness of approximately 0.54 ± 0.01 mm and lumen diameter of approximately 4.98 ± 0.01 mm. B. Phantom B with wall thickness of approximately 1.06 ± 0.01 mm and lumen diameter of approximately 5.98 ± 0.01 mm. C. Phantom C with wall thickness of approximately 1.56 ± 0.01 mm and lumen diameter of approximately 4.98 ± 0.01 mm mm. D. Human carotid artery with CIMT of 0.619 ± 0.051 mm and lumen diameter of 5.158 ± 0.034 mm. E. Human carotid artery with CIMT of 0.822 ± 0.132 mm and lumen diameter of 6.423 ± 0.072 mm. F. Human carotid artery with CIMT of 1.490 ± 0.196 mm and lumen diameter of 5.425 ± 0.090 mm. All three phantom images were recorded in water at a temperature of 38 ± 1°C with the ultrasound probe 20 mm from the leading edge of the far wall of the phantom, a DR of 55 dB and gain of 0 dB. All human images were recorded in vivo.

The B-ultrasound images were recorded using a 10 MHz multi-frequency linear array probe attached to a high-resolution ultrasound machine (Acuson Aspen, Mountain View, CA). The analogue video output from the ultrasound machine was converted into a digital DICOM 3.0 file by proprietary DICOM Encoder software. The DICOM files were recorded onto the hard-drive of a standard personal computer running Windows 2000. Figure 3 shows typical B-mode images of the 3 different phantom arteries compared with images of human arteries with similar dimensions.

### Lumen and wall-thickness measurements

**Figure 4:**
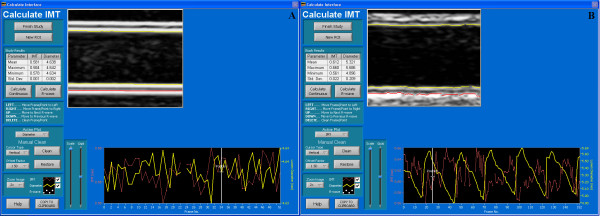
**Software used to measure lumen diameter and wall-thickness**. Screen capture of the CIMT measurement software showing the detected lumen margins (yellow lines) and the wall thickness (red line) on a magnified region of interest from an image of Phantom C (Panel A) and a human artery with a similar lumen diameter and CIMT (Panel B). The mean, maximum, minimum and standard deviations of wall thickness (IMT) and lumen (Diameter) measurements are shown in the Study Results table. The individual measurements on each of the 44 frames selected for analysis are shown in the data table below the image (IMT in red and lumen in yellow). The effect of the cardiac cycle on lumen diameter is clearly seen in the data table for the human artery measurements (Panel B).

We measured arterial lumen diameter and wall-thickness using our own CIMT measurement software, described in detail in a previous publication [13]. Briefly, the user opens a selected DICOM file in the software, chooses a single frame or multiple image frames for analysis and draws a rectangular region of interest (ROI) over the image that includes both walls of the artery. The software then uses an edge-detection algorithm to find the near and far wall lumen edges and the far-wall media-adventitia interface within the chosen ROI on all the frames selected by the user. The software marks the interfaces and calculates lumen diameter and the intima-medial thickness (Figure 4).

### Statistics

We used simple linear regression to test the univariate effects of probe distance, DR and gain on the lumen and wall-thickness measurements and a generalised linear model (GLM) to test for interactions between probe distance, DR, gain and arterial wall thickness. We used Minitab (Version 14.2, Minitab Inc, USA) for the statistical analyses.

## Results

The actual diameters of the molds, measured with a micrometer accurate to ± 0.01 mm, were 4.98 mm and 5.98 mm for the male parts and 6.06 mm and 8.09 mm for the female parts. The actual wall thicknesses and lumen diameters of the phantoms were therefore A: 0.54 ± 0.01 mm and 4.98 ± 0.01 mm, B:1.06 ± 0.01 mm and 5.98 mm, C: 1.56 ± 0.01 mm and 4.98 ± 0.01 mm respectively. We found that the moisture content of the phantom affected the wall thickness and lumen diameter so we were unable to test the absolute accuracy of our measurement software [Additional File 1]. The dimensions of phantom changed with immersion time, but the changes occurred slowly (an increase of approximately 0.02 ± 0.002 mm per hour in lumen diameter and 0.003 ± 0.003 mm per hour in wall thickness). The model remained valid for testing the effects of DR, gain and probe distance on measured lumen diameter and wall thickness, as these settings were adjusted over much shorter time intervals.

### Lumen measurements

**Figure 5:**
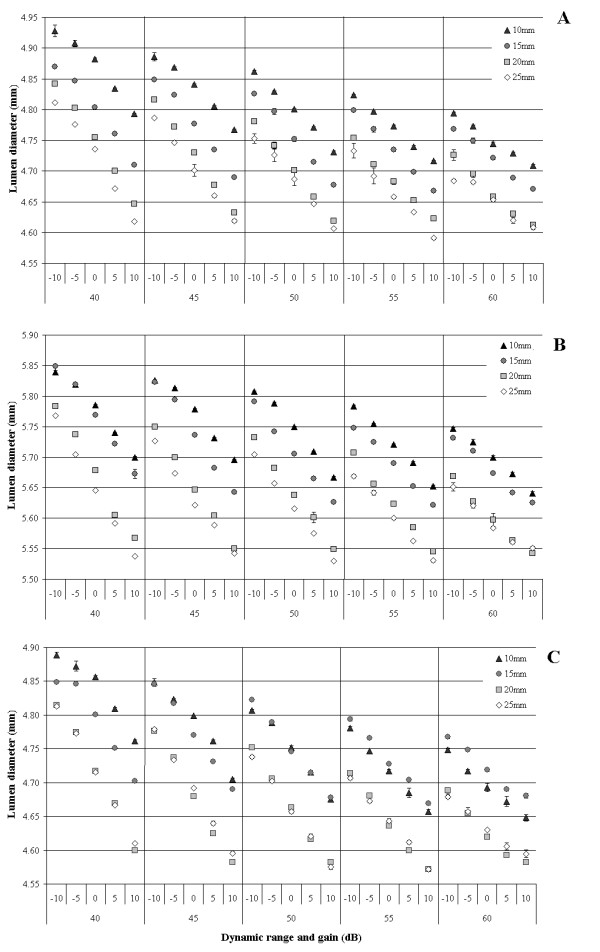
**Effect of dynamic range, gain and probe distance on measured lumen diameter**. Lumen diameter plotted against the dynamic range and gain settings used to record the images. Each data point represents the mean of 200 measurements. Error bars show standard deviation and if not visible are contained within the icon. The different symbols represent the distance between the ultrasound probe and the leading edge of the far war of the phantom artery. A. Phantom A with lumen diameter of approximately 4.98 ± 0.01 mm. B. Phantom B with lumen diameter of approximately 5.98 ± 0.01 mm. C. Phantom C with lumen diameter of approximately 4.98 ± 0.01 mm

Figure 5 shows the lumen measurements from a single phantom of each type, A, B and C. Table 1 shows the mean lumen measurements for the same phantoms at each DR, gain and distance setting. Measured lumen diameter was smaller than the actual lumen diameter and decreased in a linear manner as DR, gain and probe distance increased. For each 5 dB increase in DR there was a mean reduction in measured lumen diameter of 0.02 ± 0.004 mm (p < 0.001). For each 5 dB increase in gain there was a mean reduction in measured lumen diameter of 0.04 ± 0.004 mm (p < 0.001). For each 5 mm increase in probe distance there was a mean reduction in measured lumen diameter of 0.04 ± 0.03 mm (p < 0.001). The effect of increasing gain appeared to be greater at lower dynamic range settings and GLM analysis confirmed that the interaction between these variables was significant (p < 0.001).

**Table 1:** Effect of dynamic range, gain and probe distance on lumen diameter measurements

		**Phantom A**		**Phantom B**		**Phantom C**	
		Lumen (mm)	p	Lumen (mm)	p	Lumen (mm)	p
**Dynamic range (dB)**	*40*	4.77 ± 0.09	<0.001	5.71 ± 0.09	<0.001	4.75 ± 0.10	<0.001
	*45*	4.75 ± 0.09		5.69 ± 0.09		4.73 ± 0.10	
	*50*	4.73 ± 0.08		5.68 ± 0.08		4.71 ± 0.09	
	*55*	4.71 ± 0.08		5.66 ± 0.07		4.68 ± 0.09	
	*60*	4.70 ± 0.07		5.64 ± 0.06		4.67 ± 0.08	
							
**Gain (dB)**	*-10*	4.79 ± 0.07	<0.001	5.75 ± 0.06	<0.001	4.77 ± 0.07	<0.001
	*-5*	4.77 ± 0.07		5.71 ± 0.06		4.75 ± 0.08	
	*0*	4.74 ± 0.08		5.68 ± 0.06		4.71 ± 0.08	
	*+5*	4.70 ± 0.08		5.64 ± 0.06		4.67 ± 0.09	
	*+10*	4.67 ± 0.08		5.60 ± 0.06		4.64 ± 0.09	
							
**Probe distance (mm)**	*10*	4.80 ± 0.07	<0.001	5.74 ± 0.06	<0.001	4.75 ± 0.08	<0.001
	*15*	4.75 ± 0.08		5.71 ± 0.07		4.75 ± 0.07	
	*20*	4.70 ± 0.09		5.64 ± 0.07		4.67 ± 0.10	
	*25*	4.68 ± 0.08		5.62 ± 0.06		4.67 ± 0.10	

Data are mean ± standard deviation

n = 80 per level for dynamic range (5 gain levels × 4 distance levels × 4 angles)

n = 80 per level for gain (5 dynamic range levels × 4 distance levels × 4 angles)

n = 100 per level for probe distance (5 gain levels × 5 dynamic range levels × 4 angles)

P-values are for trend from a univariate linear regression model

Phantom A and C have an estimated lumen diameter of 4.98 ± 0.01 mm

Phantom B has an estimated lumen diameter of 5.98 ± 0.01 mm.

### Wall thickness measurements

**Figure 6:**
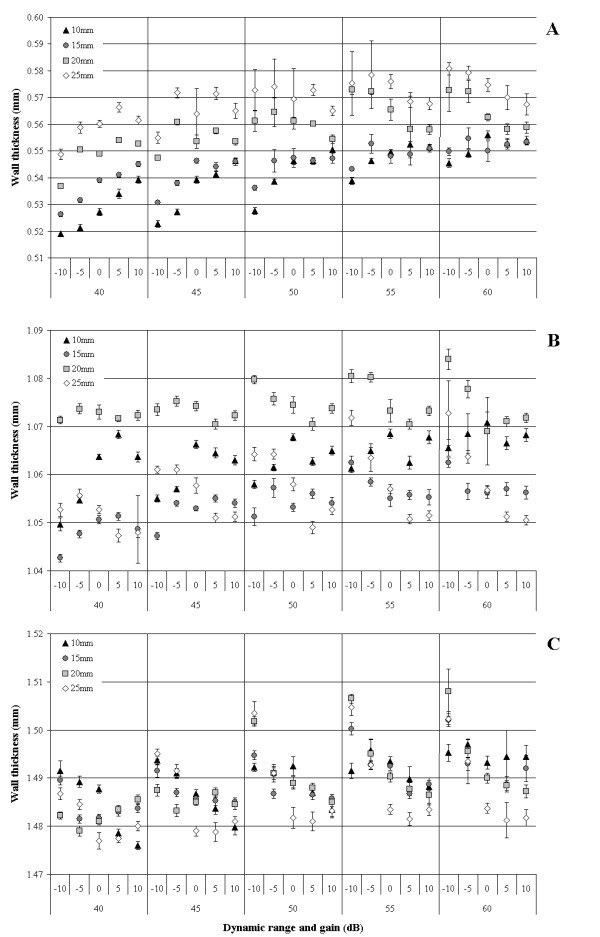
**Effect of dynamic range, gain and probe distance on measured wall-thickness**. Wall thickness plotted against the dynamic range and gain settings used to record the images. The different symbols represent the distance between the ultrasound probe and the leading edge of the far war of the phantom artery. Each data point is the mean of 200 measurements (50 frames × 4 angles). Error bars show standard deviation and if not visible are contained within the icon. A. Phantom A with wall-thickness of approximately 0.54 ± 0.01 mm. B. Phantom B with wall-thickness of approximately 1.06 ± 0.01 mm. C. Phantom C with wall-thickness of approximately 1.56 ± 0.01 mm

Figure 6 shows the wall thickness measurements for the same phantoms in which lumen diameter was assessed. Table 2 shows the average wall thickness measurement for each DR, gain and distance setting. In contrast to the lumen measurements there was no immediately obvious pattern in the effects of DR, gain or probe distance on wall thickness measurements. Regression analysis showed that DR had a consistent effect on measured wall thickness, with a mean increase of 0.003 ± 0.002 mm for each 5 dB increase in DR (p < 0.001). The effects of increasing gain and probe distance on measured wall thickness were not consistent and appeared to vary depending on the actual wall thickness of the artery.

**Table 2:** Effect of dynamic range, gain and probe distance on wall thickness measurements

		**Phantom A**		**Phantom B**		**Phantom C**	
		Wall thickness (mm)	p	Wall thickness (mm)	p	Wall thickness (mm)	p
**Dynamic range (dB)**	*40*	0.543 ± 0.042	0.002	1.058 ± 0.014	<0.001	1.483 ± 0.019	<0.001
	*45*	0.549 ± 0.041		1.061 ± 0.012		1.486 ± 0.019	
	*50*	0.554 ± 0.041		1.063 ± 0.012		1.489 ± 0.020	
	*55*	0.559 ± 0.041		1.064 ± 0.012		1.492 ± 0.020	
	*60*	0.560 ± 0.042		1.065 ± 0.013		1.493 ± 0.020	
							
**Gain (dB)**	*-10*	0.548 ± 0.044	0.38	1.064 ± 0.014	0.03	1.496 ± 0.021	<0.001
	*-5*	0.554 ± 0.043		1.064 ± 0.012		1.490 ± 0.020	
	*0*	0.554 ± 0.041		1.063 ± 0.012		1.487 ± 0.019	
	*+5*	0.555 ± 0.040		1.060 ± 0.012		1.485 ± 0.018	
	*+10*	0.554 ± 0.041		1.061 ± 0.012		1.485 ± 0.018	
							
**Probe distance (mm)**	*10*	0.541 ± 0.040	<0.001	1.064 ± 0.010	0.98	1.490 ± 0.018	0.29
	*15*	0.545 ± 0.041		1.054 ± 0.010		1.489 ± 0.015	
	*20*	0.559 ± 0.038		1.074 ± 0.008		1.489 ± 0.021	
	*25*	0.569 ± 0.042		1.057 ± 0.012		1.486 ± 0.022	

Data are mean ± standard deviation

n = 80 per level for dynamic range (5 gain levels × 4 distance levels × 4 angles)

n = 80 per level for gain (5 dynamic range levels × 4 distance levels × 4 angles)

n = 100 per level for probe distance (5 gain levels × 5 dynamic range levels × 4 angles)

P-values are for trend from a univariate linear regression model

Phantom A has an estimated mean wall thickness of 0.54 ± 0.01 mm.

Phantom B has an estimated mean wall thickness of 1.06 ± 0.01 mm.

Phantom C has an estimated mean wall thickness of 1.56 ± 0.01 mm

## Discussion

Our results show that the ultrasound settings of DR and gain significantly alter lumen diameter and arterial wall thickness measurements made with image analysis software. Lumen diameter measurements are more sensitive to changes in DR, gain and probe distance than wall thickness measurements.

The leading edge of an ultrasound echo line (the edge nearest the ultrasound probe) represents the precise location of the boundary between two tissues with different acoustic properties. The trailing edge of an echo line (the edge furthest from the ultrasound probe) does not represent any anatomical structure and is, in effect, an acoustic "shadow" cast by the tissue interface. The width of the echo line is determined by the acoustic properties of the tissues and the ultrasound settings used to record the image. The true dimensions of a structure can be estimated accurately only by measuring the distance between the leading edges of two echo lines [19].

**Figure 7:**
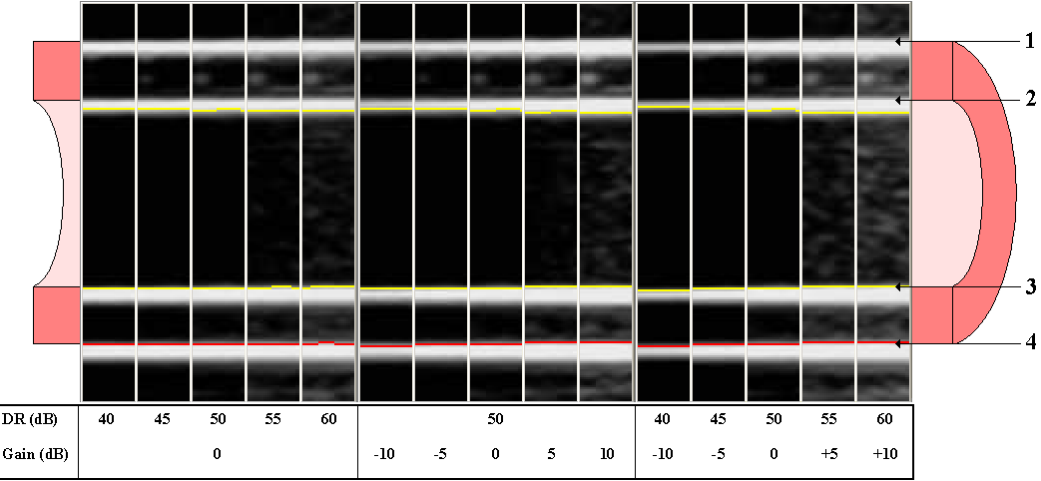
**The effect of dynamic range and gain on B-mode images of the phantom artery**. This figure shows a series of B-mode images taken from a single scan of Phantom C at a distance of 20 mm from the probe. The same region of interest was analysed to show the effect of varying dynamic range and gain settings on the appearance of the B-mode image and also on the detected interfaces. The diagram of a cross-section of a phantom artery shows how the leading edges of the near and far wall echo lines represent the interface between the agar compound and the water surrounding the artery. 1. Leading edge of near wall water-agar interface. 2. Leading edge of near wall agar-water interface. 3. Leading edge of far wall water-agar interface. 4. Leading edge of far wall agar-water interface.

Ultrasound images of phantom arteries with walls of a single layer show the same characteristic double echo line as B-mode images of real arteries (Figure 3). The leading edge of the first echo in the phantom image is generated as the ultrasound beam enters the agar from the water and the second echo as the beam exits the agar back into the water (Figure 7). The distance from the leading edge of the first echo line to the leading edge of the second echo line thus represents the thickness of the phantom artery wall and, if measured on the far wall, simulates CIMT measurement in a real artery (Figure 4).

Despite problems with the agar compound absorbing water, the measured wall thickness for phantoms A and B closely approximated the expected wall thickness. However, the measured wall thickness of phantom C was significantly less than the expected value (Table 2). The most likely explanation for the discrepancy is that the male part of the mold was not accurately centred in the female part when this phantom was made. We found that phantoms constructed with this particular combination of male and female parts tended to have walls of unequal thickness [Additional File 2]. Although we tried to compensate for the asymmetry by rotating the artery a quarter turn between scans, it seems likely that this particular phantom was scanned more frequently with a thinner far wall than a thick far wall. It is also possible that the artery had shrunk due to dehydration, although this seems less likely as all the phantoms were constructed the evening before they were scanned and were extracted straight from the mold into the water bath.

By contrast with the general accuracy of the wall thickness measurements, lumen measurements significantly underestimated the actual diameter (Table 1), a phenomenon that has been reported previously with intravascular ultrasound measurements [15]. The main reason for the inaccuracy in our study is that our image analysis software, like the majority of available systems, measures lumen diameter from the *trailing *edge of the near wall intima-lumen echo and not the leading edge (Figures 4 and 7). Ideally, lumen diameter should be measured from the leading edge of the near wall lumen-intima interface [19], but this is more technically difficult than measuring from the trailing edge and is rarely done in practice. First, the brachial and carotid arteries are mobile and superficial vessels, which can make it difficult to obtain and keep a clear image of the narrow near-wall intima-lumen echo-line. Second, it is simpler to program an edge-detecting algorithm that tracks upwards from a dark lumen to find the first bright near-wall echo line than to program an algorithm that reliably detects the top edge of a narrow echo line that frequently fades in and out of focus from frame to frame. The former type of algorithm will generally find and track an arterial margin, even if it is the trailing edge of the near-wall adventitia-media echo.

Figure 7 shows how adjusting the gain and DR settings altered the detected echo interfaces. An increase in gain of 5 dB reduced measured lumen diameter by approximately 0.04 mm and in DR by approximately 0.02 mm. The brachial artery has an average internal diameter of less than 4 mm in most humans, so a change of 0.04 mm represents difference in measured lumen diameter that is greater than or equal to 1%. Between-group differences of this magnitude in the maximum post-ischemia brachial artery diameter are frequently reported in the literature as being statistically and clinically significant [20-22].

We found that the distance between the probe and the artery also affected lumen measurements. For a given DR and gain setting, a 5 mm change in probe distance causing a 0.04 mm difference in measured lumen. It is possible that this effect was an artefact of the phantom model and, as such, may not translate to ultrasound scans recorded in vivo. However, our results should be kept in mind when interpreting the reported effects of obesity, weight loss or weight training on arterial diameters.

Although the leading rather than trailing edges of the far wall echo lines were used to assess wall thickness, DR and gain also appeared to affect these measurements, although to a lesser degree than lumen diameter. Each 5 dB increment in DR increased measured wall thickness by 0.003 mm. This error might appear trivial, but CIMT progresses very slowly in most people, at the rate of 0.001 mm to 0.03 mm per year, and reductions in mean CIMT of this magnitude have been reported as significant after cholesterol-lowering treatment [23-25]. Increasing gain also appeared to reduce measured wall thickness, particularly in the thick-walled phantoms, but there was no clear or consistent pattern. Similarly there was no consistent effect of probe distance on measured wall thickness. The apparent differences due to distance in Figure 6 were probably caused by slight alterations in the angle of insonation when the probe was moved relative to the phantom. We tried to maintain the same probe position for all scans but without clear landmarks in the phantom images it was impossible to ensure that this was actually the case. A slight random change in the angle of insonation would have affected measured wall thickness to a greater degree than lumen diameter as a result of the asymmetry discussed above, and would thus account for the apparently inconsistent effects of changing the probe distance.

Consensus guidelines for the ultrasound assessment of FMD published in 2002 make recommendations regarding subject preparation, equipment, image acquisition and analysis [26]. These guidelines suggest that sonographers should document "scan-factors", but fail to clarify what these factors should be. Our data indicate that sonographers should document the DR and overall gain settings used to record the scans and ensure that the same settings are used for baseline and post-intervention measurements. Our results also underline the importance of not altering the DR and gain settings while the ultrasound images are recorded, particularly between the baseline and post-ischemic stimulus image sequences.

Recently published consensus guidelines for the measurement of CIMT recommend that "log gain compensation *(dynamic range) *should be around 60 dB" [14]. The authors state that the lumen of the carotid should also be measured as CIMT is significantly correlated with arterial diameter. Our results support their recommendation of a relatively high DR setting when assessing lumen diameter and CIMT. CIMT measurements are less sensitive to changes in overall gain at a high DR. However, investigators should be aware that lumen diameter is underestimated by a greater amount when DR is high than when it is low.

One of the limitations of our study is that we did not assess the effect of altering the depth gain compensation (DGC) on lumen and CIMT measurements. DGC compensates for the attenuation of the acoustic signal due to absorption, scatter, and reflection. When lumen and CIMT are measured with image analysis software, DGC is commonly used to selectively brighten the arterial walls and to darken the centre of the lumen. Although we adjusted the DGC in this manner on each test image of the phantom, with the DR set at 50 dB and an overall gain of 0 dB, we did not adjust the DGC again to compensate for the subsequent changes in overall gain or DR. It is quite possible that adjusting the DGC would have attenuated the effect that these settings had on measured lumen diameter and wall thickness.

Another potential limitation of the study is that we used phantom arteries rather than real arteries to test the effect of altering ultrasound settings. However, an agar phantom in water at 37°C has very similar acoustic properties to human tissue, so there is no reason to assume that our results would not be similar in vivo. Using phantoms also provided us with arteries of known wall thickness and lumen diameter and gave us the advantage of controlling some variables that couldn't be controlled in human subjects. We have collected some preliminary data demonstrating that lumen and CIMT measurements in human subjects are affected in exactly the way predicted by the phantom results (unpublished data), but other investigators may wish to confirm our results in a larger sample.

## Conclusion

DR, gain and probe distance significantly alter lumen diameter and CIMT measurements made using image analysis software. When CIMT and FMD are used to test the efficacy of cardioprotective interventions, the DR, gain and probe position used to record baseline scans should be documented and replicated in post-treatment scans in individual trial subjects. If more than one sonographer or imaging centre is used to collect data, the study protocol should document specific DR and gain settings to be used in all subjects.

## Competing interests

The author(s) declare that they have no competing interests.

## Authors' contributions

KP designed the study, constructed and scanned the phantoms, analysed the data and wrote the manuscript. CR wrote the CIMT measurement software, assisted with data analysis and critically reviewed the manuscript. DJG, GJH and LFA critically reviewed the manuscript. All authors have read and approved the final manuscript.

## Supplementary Material

Additional file 1 Effect of time in water bath on agar phantom dimensions. Excel spreadsheet containing raw data and graphs of lumen diameter and wall thickness in two agar phantoms of type A and C measured over time in the water bath. Click here for file

Additional file 2 Effect of angle of insonation on measured wall thickness of agar phantom. Excel spreadsheet containing raw data and graphs of lumen diameter and wall thickness in an agar phantom of type C. Test of each of four marked positions on measured IMT and lumen in a phantom artery of type C. A and C diagonally opposite and B and D diagonally opposite and perpendicular to A_C. Phantom turned through 90 degrees for each scan. Probe and settings left unchanged. Probe at 1.42 cm from leading edge of near wall of phantom. ROI optimised for analysis where possible (changed when bubbles in wall would affect analysis). Average of 49 frames analysed for each setting. Click here for file
